# Robot-Assisted Laparoscopic Removal of a Large Primary Retroperitoneal Mature Cystic Teratoma in an Adult

**DOI:** 10.7759/cureus.16329

**Published:** 2021-07-12

**Authors:** Andrew Blazek, Benjamin Plambeck, Subodh Lele, Brett C Hill

**Affiliations:** 1 Division of Urologic Surgery, University of Nebraska Medical Center, Omaha, USA; 2 Department of Pathology, University of Nebraska Medical Center, Omaha, USA

**Keywords:** primary retroperitoneal teratoma, cystic retroperitoneal mass, robot assisted surgery, benign mature cystic teratoma, laparoscopic resection

## Abstract

Mature teratomas are unique and generally benign neoplasms. They are derived from embryonic tissues and typically located within the gonadal region. Primary retroperitoneal teratomas are uncommon in adults and often challenging to treat, given their location and size. Here, we offer a rare case of a large primary retroperitoneal mature cystic teratoma, detected on abdominal ultrasound during the work-up of abdominal bloating and nausea and treated with robot-assisted laparoscopic excision in a 58-year-old male. In this report, we sought to describe the evaluation, treatment, and follow-up of this condition, as well as review the associated literature.

## Introduction

Within the realm of neoplasms, teratomas are a rarity and consist of two or more cell types from the three germ layers (endoderm, mesoderm, or ectoderm) [1]. Teratomas tend to occur in the gonads [2]; however, when extragonadal, they can occur at midline structures within the head, neck, mediastinum, sacrococcygeal regions, and retroperitoneum [1-5]. Primary retroperitoneal teratomas, which arise much more commonly in children, make up only 4% of all primary teratomas [6]. In this report, we present a rare case of a large primary retroperitoneal mature cystic teratoma in a 58-year-old male, successfully managed with robot-assisted laparoscopic removal. 

## Case presentation

**Figure 1:**
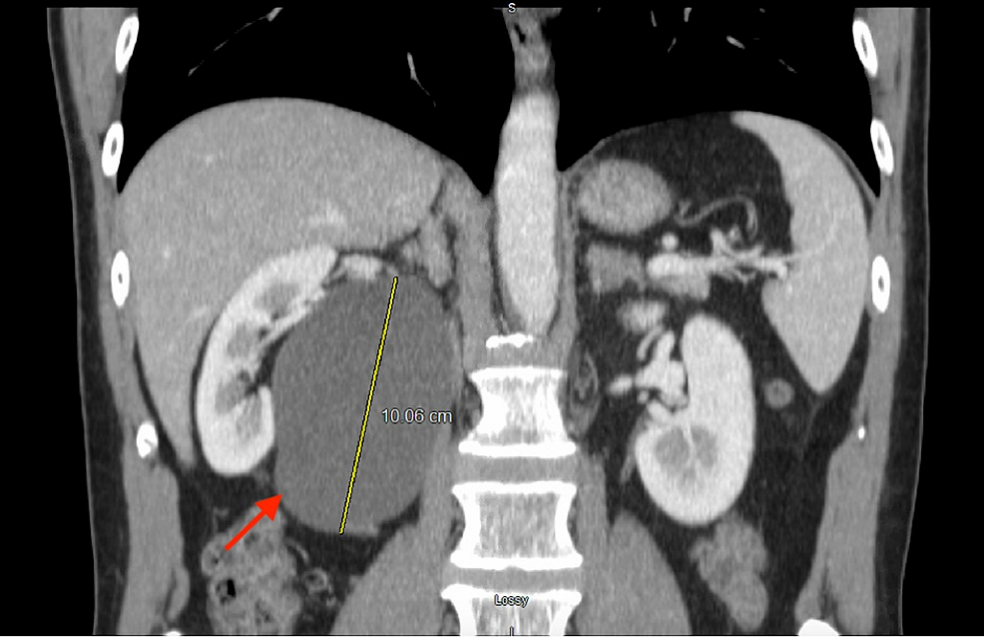
Coronal CT scan image showing a cystic mass (red arrow) adjacent to the right kidney measuring approximately 10.1 cm in craniocaudal dimension. CT: computed tomography

**Figure 2:**
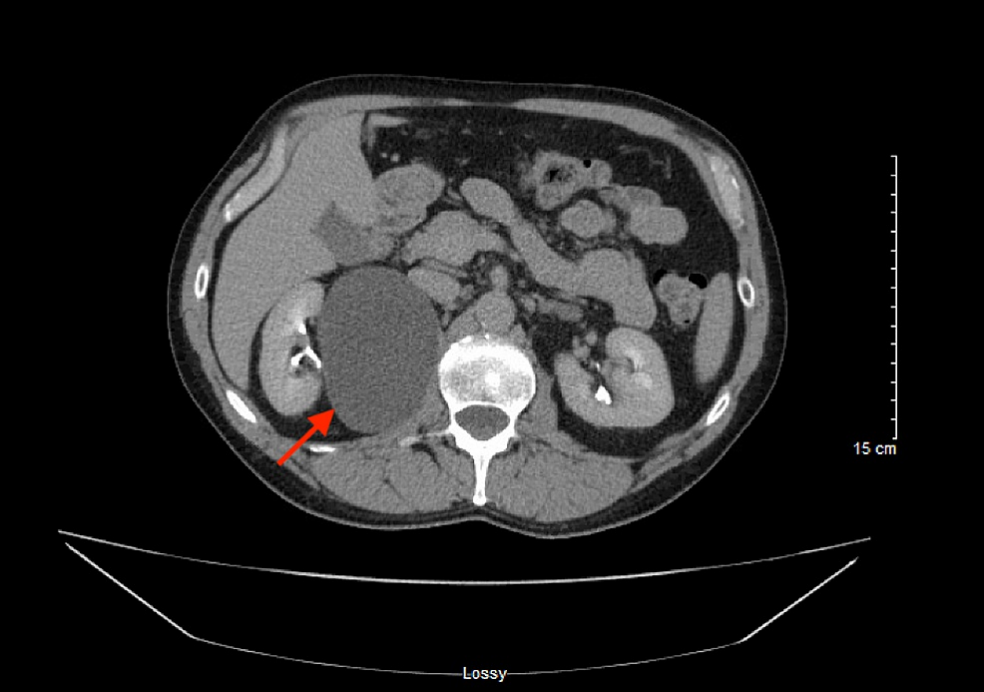
Axial CT scan image showing a cystic mass (red arrow) at the medial aspect of the right kidney measuring 8.8 cm in anterior-posterior dimension and 7 cm in medial-lateral dimension. CT: computed tomography

A 58-year-old male initially presented to his primary care provider for evaluation of abdominal bloating, early satiety, nausea/vomiting, gastroesophageal reflux, and occasional intermittent flank pain. The patient was referred to gastroenterology, and an esophagogastroduodenoscopy (EGD) and colonoscopy with biopsies revealed chronic inflammation on pathology. As the patient had no improvement on medical therapy, an abdominal ultrasound was obtained which revealed a large renal cyst. No other abnormalities were noted at that time. A CT abdomen and pelvis with and without contrast was obtained, revealing what appeared to be a large exophytic right renal cyst measuring 10.1 x 8.8 x 7.0 cm (Figures 1, 2). The patient was referred to urology for further evaluation of these findings. 

The patient had a past medical history significant for a left, non-toxic thyroid nodule discovered on ultrasound in February 2021. He had no history of abdominal surgery or genitourinary operations. The patient did not have any family history of genitourinary malignancies or other significant cancer history. At initial presentation, vital signs and physical exam were normal. There were no abdominal masses and his genitourinary exam was negative for scrotal masses. Prior to surgery, his creatinine was 0.92 mg/dL. No tumor markers were drawn, and the patient was scheduled for a right robot-assisted laparoscopic cyst excision/decortication.

Right robot-assisted laparoscopic cyst excision began in the typical fashion. Upon encountering the cystic mass, it was noted to have an abnormal tan/brown appearance. In addition, the mass appeared to originate from the retroperitoneum rather than the right kidney, although it significantly displaced the kidney anteriorly. As we proceeded with our dissection, the kidney was noted to have both a superior and inferior vascular supply. The inferior renal artery/vein was significantly distended and unable to be easily mobilized, inhibiting our dissection. Thus, the inferior vascular supply was ligated in order to complete tumor removal. Overall, the dissection was difficult due to the anterior displacement of the right kidney and significant distention of the inferior renal vessels. Once the mass was free from adjacent structures, it was placed into an Endocatch bag and removed. As the bag was drawn through the incision, the cyst wall of the mass ruptured and a thick, yellow fluid with hair was noted. In the end, we performed a right robot-assisted excision of a cystic retroperitoneal mass.

**Figure 3:**
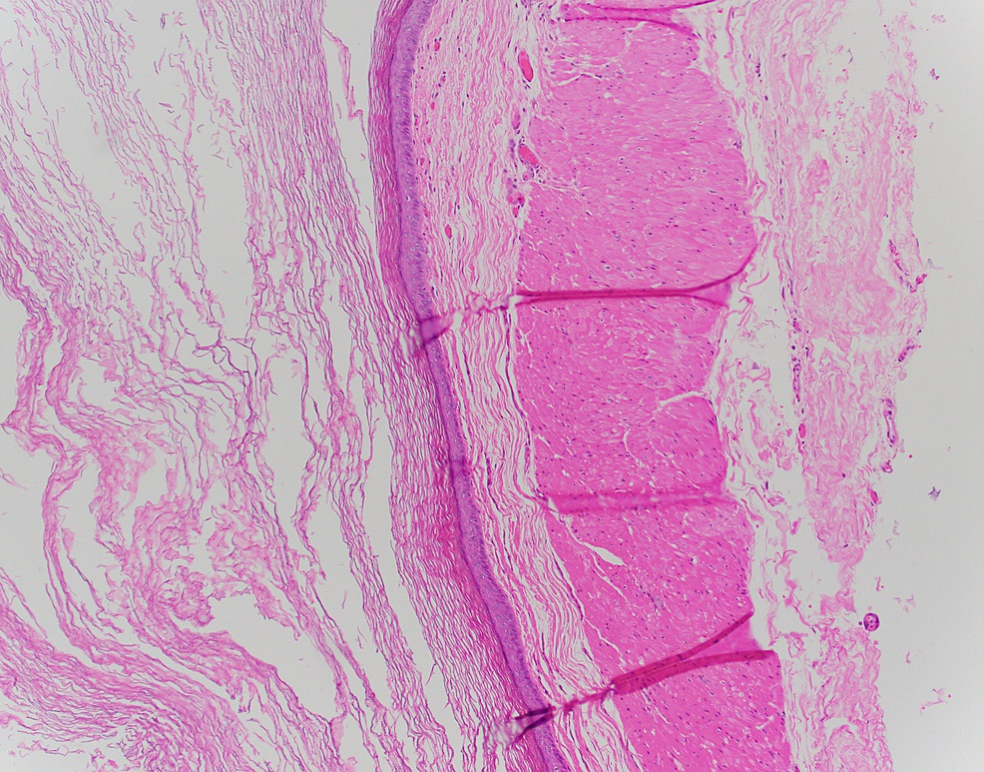
Histopathologic image of the cystic teratoma wall (40x). Image shows keratinized epithelium with smooth muscle below the epithelium. Findings are consistent with the diagnosis of a cystic teratoma.

The patient tolerated surgery well and discharged on post-operative day 1. After surgery, his creatinine was 0.95 mg/dL, and he was followed closely for two weeks with no significant complications or complaints. Pathology assessment revealed a cyst wall including focal smooth muscle and squamous epithelium with keratinization (Figure 3). The final diagnosis was a mature cystic teratoma with negative margins and no evidence of malignancy or immature components. A scrotal ultrasound, which was obtained due to the abnormal pathology, was normal, and the patient was doing excellent at his two-month follow-up.

## Discussion

Teratomas are masses composed of two or more cell types from the three germ layers and may contain any tissue type. Cystic teratomas may contain fully mature elements, such as hair and sebaceous tissue, and they are typically benign compared to their solid counterparts. These neoplasms most commonly arise from the gonads and sacrococcygeal region; but in rare instances, they can arise from the retroperitoneum. The incidence of retroperitoneal teratomas peaks once during the first year of life (43%-55%) and again in early adulthood. Primary retroperitoneal teratomas are exceedingly rare tumors in adults, with few cases presented in the literature [1]. Research shows that fewer than 10%-20% of retroperitoneal teratomas arise in adults above 30 years old [7], and when they do occur, they predominantly originate from the left retroperitoneum in a suprarenal location [2,5,8]. Our case is a very rare entity, given the right-sided and retroperitoneal location of the tumor, the age of our patient (58 years old), and removal of the large primary retroperitoneal teratoma using robot-assisted laparoscopic excision.

Benign teratomas are typically asymptomatic and often incidental findings on imaging; however, as these tumors increase in size, various obstructive symptoms can develop. These include back pain, abdominal pain, gastrointestinal symptoms, genitourinary symptoms, lower extremity swelling, and abdominal fullness. In addition, a midline abdominal mass may be palpated on exam [9]. In our case, the patient presented with obstructive gastrointestinal and genitourinary symptoms.

As it relates to diagnosis for these masses, radiographic assessment is essential to pre-operative evaluation. Historically, X-rays helped distinguish between calcified components of teratomas; however, ultrasound and CT scans are now used to provide extra information on the mass and its constituents [1,2]. MRI is the superior technique for evaluating these tumors, as it provides exceptional soft tissue details, precisely identifies malignant portions of the mass, and aids in tumor staging [1,10]. However, histological analysis of a surgically obtained specimen is ultimately required for definitive diagnosis [1]. 

Surgical resection is critical to both the diagnosis and treatment of these tumors, whereas radiation and chemotherapy play minor roles in the overall treatment strategy for benign teratomas [1,11]. However, these operations can become difficult due to tumor location and nearby vital structures. Laparoscopic resection of retroperitoneal teratomas has been described and is supported in the literature for benign tumors [1], as it offers superior procedure morbidity, recovery, and operation time [12]. A robotic approach has its advantage as well, as it can share similar peri-operative complications as standard laparoscopy, while enhancing the ability to remove more surgically complex disease [13]. Robotic surgery has been well-described as an effective technique for procedures such as retroperitoneal lymph node dissections (RPLND) [14]; however, despite frequent utilization of robots in urology, robot-assisted laparoscopic excision of primary retroperitoneal tumors is not well-documented, with fewer than 45 cases reported in the literature [15]. Furthermore, primary retroperitoneal teratomas make up only 1-11% of this larger group of retroperitoneal tumors [1], indicating that robotic surgery is even less frequently used for these tumors. To our knowledge, this is the first reported case of robot-assisted laparoscopic excision for a large (> 10 cm) primary retroperitoneal cystic teratoma, initially presenting in a 58-year-old adult.

Survival of patients with primary retroperitoneal teratomas is best when surgically managed, with virtually 100% survival rates after five years for benign teratomas [16]. After surgery, disease-free survival is dependent on the extent of tumor removal, and, thus, surgical resection in low-volume disease is beneficial [17]. Malignant transformation of a mature teratoma to a sarcoma or carcinoma can occur in 3%-6% of patients [17-18]. Therefore, residual disease after surgery may lead to relapse, and patients should be monitored with an annual CT scan to detect asymptomatic progression/recurrence [17]. 

## Conclusions

Primary retroperitoneal teratomas are extremely rare tumors in adults. They are usually asymptomatic and incidentally found but may present with abdominal symptoms if large enough. While the diagnosis can be implicated preoperatively on imaging, definitive diagnosis and treatment both require surgical resection of the tumor. Robot-assisted laparoscopic excision is a safe and effective method of surgical management for large primary retroperitoneal mature cystic teratomas. Finally, patients should undergo yearly follow-up for detection of recurrence.
